# Use of high throughput qPCR screening to rapidly clone low frequency tumour specific T-cells from peripheral blood for adoptive immunotherapy

**DOI:** 10.1186/1479-5876-6-60

**Published:** 2008-10-20

**Authors:** Udai S Kammula, Oscar K Serrano

**Affiliations:** 1 Surgery Branch, National Cancer Institute, Bethesda, MD, USA

## Abstract

**Background:**

The adoptive transfer of autologous tumor reactive lymphocytes can mediate significant tumor regression in some patients with refractory metastatic cancer. However, a significant obstacle for this promising therapy has been the availability of highly efficient methods to rapidly isolate and expand a variety of potentially rare tumor reactive lymphocytes from the natural repertoire of cancer patients.

**Methods:**

We developed a novel *in vitro *T cell cloning methodology using high throughput quantitative RT-PCR (qPCR assay) as a rapid functional screen to detect and facilitate the limiting dilution cloning of a variety of low frequency T cells from bulk PBMC. In preclinical studies, this strategy was applied to the isolation and expansion of gp100 specific CD8+ T cell clones from the peripheral blood of melanoma patients.

**Results:**

In optimization studies, the qPCR assay could detect the reactivity of 1 antigen specific T cell in 100,000 background cells. When applied to short term sensitized PBMC microcultures, this assay could detect T cell reactivity against a variety of known melanoma tumor epitopes. This screening was combined with early limiting dilution cloning to rapidly isolate gp100_154–162 _reactive CD8+ T cell clones. These clones were highly avid against peptide pulsed targets and melanoma tumor lines. They had an effector memory phenotype and showed significant proliferative capacity to reach cell numbers appropriate for adoptive transfer trials (~10^10 ^cells).

**Conclusion:**

This report describes a novel high efficiency strategy to clone tumor reactive T cells from peripheral blood for use in adoptive immunotherapy.

## Background

Adoptive immunotherapy with autologous tumor infiltrating lymphocytes (TIL) in conjunction with a lymphodepleting conditioning regimen can mediate significant tumor regression in ~50% of patients with refractory metastatic melanoma [1,2]. However, not all patients with melanoma are eligible for this type of immunotherapy either because resectable tumor is not available, the lymphocytes from the specimen do not expand sufficiently, or the lymphocytes that do proliferate do not exhibit sufficient tumor specific reactivity. When reactive polyclonal TIL are generated, the composition is largely unknown and can vary significantly between patients. Thus, it has been difficult from these pilot clinical studies to define an "optimal" population of lymphocytes suitable to consistently mediate tumor regression in melanoma patients. We hypothesized that an iterative strategy of isolation, transfer, and clinical evaluation of a series of autologous tumor reactive lymphocyte clones would help to define the antigenic targets and lymphocyte characteristics associated with therapeutic efficacy for future adoptive immunotherapy efforts. Further, we hypothesized that isolation of these lymphocytes from peripheral blood (PBL) would have several advantages. From a practical perspective, procuring tumor reactive lymphocytes from a blood draw or leukapheresis would avoid the need for surgery and the potential for post-operative complications and delays. The broad repertoire of PBL might allow for the isolation of unique populations of tumor reactive lymphocytes that are not commonly found in TIL. Finally, the use of PBL may serve as a generalized strategy to isolate tumor reactive lymphocytes from patients with diverse histologies and, thus, expand the therapeutic relevance of this approach.

A significant obstacle to this clinical strategy has been the availability of highly efficient *in vitro *methods to rapidly isolate and expand tumor reactive T cell clones from the peripheral repertoire. Many attractive tumor antigens are derived from normal self proteins and conventional views of immunologic tolerance suggest that T cells reactive against these self antigens are rare in the natural peripheral repertoire and are predominantly of low functional avidity, incapable of recognizing tumor cells. Not surprisingly, prior clinical trials isolating specific populations of melanoma reactive T cells from PBL have typically relied on repetitive *in vitro *antigen stimulation over extended culture periods [3-6] and/or the need for peptide/MHC multimer sorting to help enrich for low frequency T cells [7]. Further, these studies [4-6] have almost exclusively focused on the isolation of MART reactive CD8+ T cells which can naturally exist at high frequencies in the peripheral repertoire of both melanoma patients and healthy individuals [8]. The adoptive transfer of lower frequency CD8+ T cells from peripheral blood that recognize other melanoma antigens, such as gp100, has been studied in few patients [6] or has required heteroclitic peptide immunization of the host to increase the precursor frequency prior to isolation [9-11].

To overcome the significant challenge of efficiently cloning a variety of potentially rare tumor reactive lymphocytes from the natural peripheral blood repertoire of cancer patients, we developed a novel *in vitro *methodology using quantitative RT-PCR (qPCR) as a high throughput functional screen to identify low frequency T cell clones for rapid isolation. In this report we describe the development of this methodology and an example of its application in the isolation and expansion of low frequency gp100_154–162 _reactive CD8+ T cell clones from peripheral blood for use in adoptive immunotherapy clinical trials.

## Methods

### Media and cell culture

Human cultured cell lines included T2 cells (HLA-A2+ peptide transporter-associated protein deficient T-B hybrid), melanoma tumor lines (526 mel, 624 mel, 888 mel), and a hepatoma cell line (Hep 3B). All of these cell lines were routinely cultured in complete medium (CM) consisting of RPMI 1640 supplemented with 10% heat-inactivated fetal bovine serum, 2 mM L-glutamine (Invitrogen, Carlsbad, CA), 50 units/mL penicillin (Invitrogen), 50 μg/mL streptomycin (Invitrogen), 50 μg/mL gentamicin (Invitrogen), 10 mM Hepes (Invitrogen), and 250 ng/mL Amphotericin B (Invitrogen). Human lymphocytes were cultured in CM with 10% heat-inactivated human AB serum (Gemini Bio-Products, Woodland, CA). The CD8+ T cell clone, C6E4, recognizes the gp100_154–162 _epitope and was generated by limiting dilution from the peripheral blood of a patient with metastatic melanoma.

### Patient PBMC

PBMCs used in this study were obtained by leukapheresis from HLA-A2+ metastatic melanoma patients evaluated on IRB approved protocols at the Surgery Branch, National Cancer Institute (NCI, National Institutes of Health, Bethesda, MD).

### Peptides

Synthetic peptides were made using a solid phase method on a peptide synthesizer (Gilson) at the Surgery Branch (NCI). The purity of each peptide was confirmed by mass spectrometry and each was resuspended to 1 mg/ml for *in vitro *use. The sequences of the peptides used in this study are as follows: gp100_209–217 _(ITDQVPFSV), gp100_154–162_(KTWGQYWQV), MART-1_27–35 _(AAGIGILTV), HIVpol_476–484 _(ILKEPVHGV), and FLU M1_58–66 _(GILGFVFTL).

### *In vitro *sensitization of PBMC

PBMC from HLA-A2+ melanoma patients underwent *in vitro *sensitization for a total of either 6 or 10 days as follows: On day 0, cryopreserved PBMC's were thawed, washed twice with CM, and plated in a 96-well plate (3 × 10^5 ^cells/well; 0.2 mL/well). Plates were incubated at 37°C in 5%CO_2 _overnight to recover from the thaw. On day 1, the sensitizing peptide was added to the PBMC culture plate at a final concentration of 1 μg/ml. On day 2, 90 IU/ml recombinant interleukin 2 (IL-2; Chiron Co., Emeryville, CA) was added to the cultures. On day 6, the sensitized cultures were assayed for peptide reactivity by either the qPCR assay or ELISA based cytokine release assay. Alternatively, for some experiments, sensitization was performed for a total 10 days. This procedure was identical to the 6 day sensitization except that an additional peptide exposure was performed on day 6, IL-2 (90 IU/ml) was added on day 7, and the cultures assayed for reactivity on day 10.

### High throughput real-time interferon-γ qPCR assay

On the day of assay (day 6 or 10), T2 cells were pulsed separately with the relevant sensitizing peptide and an irrelevant peptide at 1 μg/ml in medium for ~2 hrs at 37°C, followed by washing to remove unbound peptide. From each bulk PBMC culture to be assayed, two equal aliquots of cells (each ~50 μl) were removed and incubated in parallel with 4 × 10^4 ^T2 cells (pulsed with either relevant or irrelevant peptides) in a 0.2-ml volume in individual wells of a 96 well U-bottom tissue culture plate. After 3 hours of incubation, the 96 well plate was spun (900 RPM, 5 minutes), the supernatant completely discarded, and the cell pellet placed in RLT lysis buffer (Qiagen, Valencia, CA). RNA isolation was performed in a 96 well format using the RNeasy 96 BioRobot 8000 kit (Qiagen). Total RNA for each sample was transcribed into complementary DNA (cDNA) using TaqMan Reverse Transcription Reagants (Applied Biosystems, Foster City, CA). Quantitative real-time PCR was performed to determine the copy number for interferon-γ (IFN-γ) mRNA in each sample, as described previously [12,13] using the ABI 7500 Fast Real-Time PCR System (Applied Biosystems, Foster City, CA). The IFN-γ mRNA levels in response to the relevant peptide was divided by the IFN-γ mRNA levels induced by the irrelevant HIVpol peptide to define a stimulation index (SI) for each parental PBMC culture: SI = IFN-γ (peptide *x*)/IFN-γ (HIV_pol_). A PBMC sample with a SI > 2 was considered as having specific peptide reactivity. All samples analyzed had C_T _values less than 35 cycles to ensure the quality of the PBMC samples in the assay.

### ELISA based cytokine release assay

PBMC and derived lymphocyte cultures were tested for antigen specific reactivity in a cytokine release assay using commercially available IFN-γ ELISA kits (Endogen). T2 cells were pulsed with peptide (1 μg/ml or as described in the figures) in medium for ~2 hrs at 37°C, followed by washing before initiation of co-cultures. For these assays, 10^5 ^responder cells (PBL or cloned T cells) and 10^5^stimulator cells (T2 cells or tumor lines) were co-incubated in a 0.2-ml volume in individual wells of a 96-well plate. Supernatants were harvested from duplicate wells after 20–24 hours and IFN-γ secretion was measured in culture supernatants diluted as to be in the linear range of the assay. All data is presented as a mean of duplicate samples. Cultures with IFN-γ production greater than 100 pg/ml and twice background were considered as having specific antigen reactivity.

### Cloning and expansion of antigen specific T cells

The individual cultures that exhibited the highest specific peptide reactivity by the qPCR assay were selected for limiting dilution cloning. Briefly, PBMC from a reactive culture were plated between 1 and 5 cells/well in 96-well U-bottom plates in 0.2 ml CM containing 30 ng/ml ortho-anti-CD3 (Ortho-Biotech, Raritan, NJ) and 300 IU/ml IL-2 with 5 × 10^4 ^allogeneic irradiated (4000 rad) PBMCs/well derived from at least 3 different donors. On day 5 and every 3–4 days thereafter, half of the media in each well was replaced with fresh media containing IL-2. Approximately 2 weeks after culture initiation, wells in which cell growth was visibly apparent were screened in a microcytotoxicity assay to identify clones with cytolytic activity against peptide pulsed T2 cells. Further characterization of clone function was performed using IFN-γ secretion (as described above) in response to limiting concentrations of peptide pulsed onto T2 cells and antigen positive tumor lines. Selected clones were rapidly expanded with 30 ng/ml ortho-anti-CD3 and 5 × 10^6 ^irradiated allogeneic PBMCs in upright 25-cm^2 ^flasks as described previously^10^. Additional rapid expansions were performed to determine proliferative capacity of clones. Expanded clones were re-evaluated for peptide and tumor recognition and cell surface phenotype by FACS.

### Tetramers, mAbs, and flow cytometric immunofluorescence analysis

Allophycocyanin-labeled gp100_209–217 _(ITDQVPFSV) peptide/HLA-A*0201 tetramer complexes were obtained from Immunotech, Beckman Coulter. Phycoerythrin-conjugated gp100_154–162 _(KTWGQYWQV) peptide/HLA-A*0201 tetramer complexes were obtained from the National Institutes of Health Tetramer Facility. FITC-conjugated anti-CD8, CD25, CD27, CD28, CD45RO, CD45RA, CD62L (L-selectin) monoclonal antibodies were obtained from BD Biosciences. Immunofluorescence, analyzed as the relative log fluorescence of live cells, was measured using a FACScan flow cytometer (BD Biosciences). A combination of forward angle light scatter and propidium iodide staining was used to gate out dead cells. Approximately 1 × 10^5 ^cells were analyzed. Cells were stained in a FACS buffer made of PBS (BioWhittaker) and 0.5% BSA.

## Results

### Development of a sensitive qPCR functional assay for the detection of low frequency antigen specific T cells in peripheral blood

**Figure 1:**
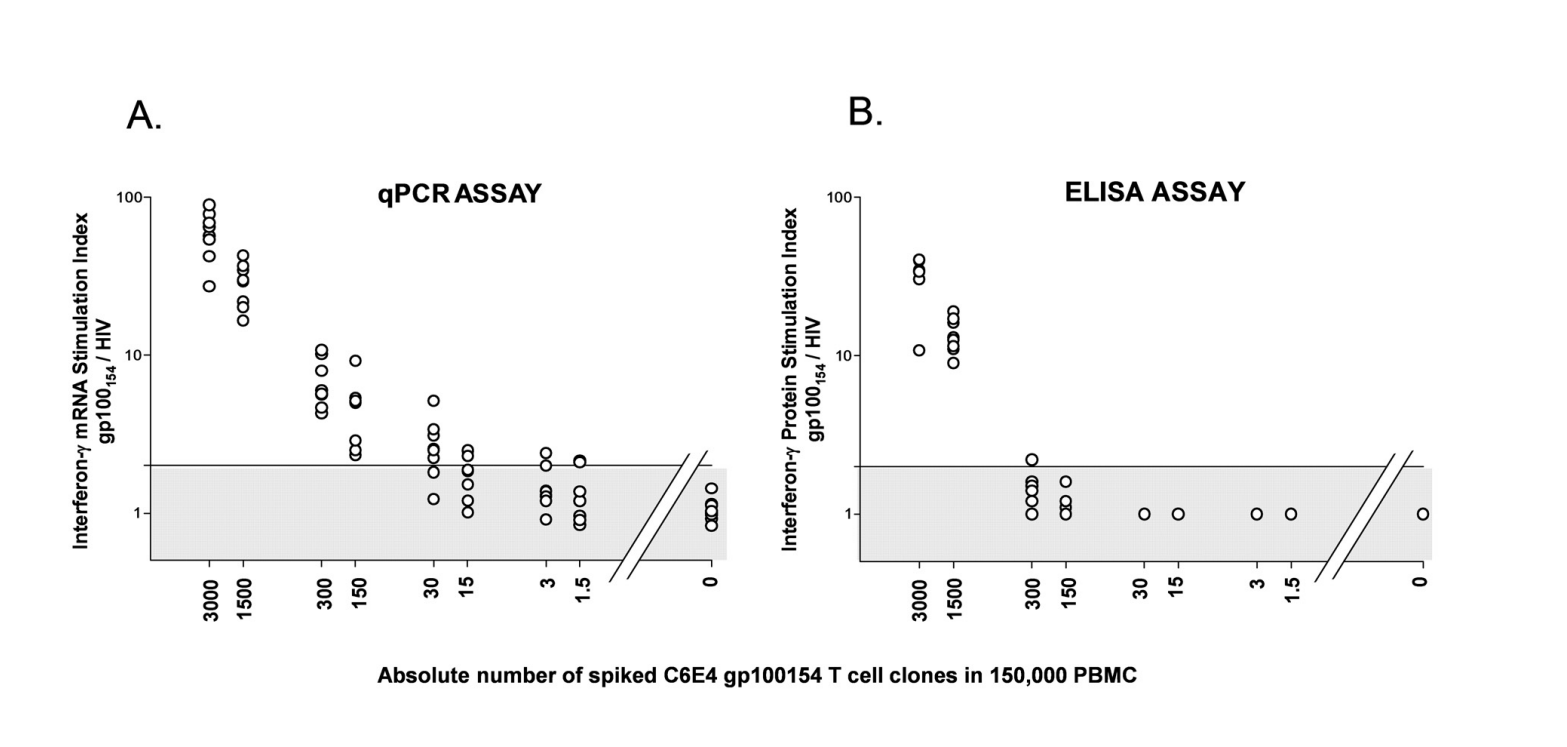
**Comparison between qPCR and ELISA based assays in the functional detection of antigen specific T cells**. Between 1.5 and 3000 gp100_154–162 _reactive CD8+ T cell clones (C6E4) were spiked into 150,000 nonreactive autologous PBMC in individual microwells (n = 8) of a 96 well plate and immediately tested for T cell recognition of T2 cells pulsed with gp100_154–162 _peptide (1 μM) and HIV_pol _peptide (1 μM). (A.) qPCR Assay. Cellular IFN-γ mRNA production was measured by qPCR at 3 hours and reported as a stimulation index (SI). SI = IFN-γ mRNA (gp100_154–162_)/IFN-γ mRNA (HIVpol). Reactive wells (SI > 2) could be identified at every dilution down to 1.5 cells spiked into 150,000 PBMC. (B.) ELISA Assay. Supernatant IFN-γ protein production was measured at 24 hours by standard ELISA. SI = IFN-γ protein (gp100_154–162_)/IFN-γ protein (HIVpol). Reactive wells (SI > 2) could be identified at dilutions down to 300 cells spiked into 150,000 PBMC. (O) represents the SI for each microwell. Shaded area represents range of non-specific reactivity (SI ≤ 2).

Sensitive detection of rare antigen specific T cells in bulk heterogeneous populations is essential to their rapid and efficient isolation. We originally described the measurement of antigen induced interferon-γ mRNA by qPCR as a highly sensitive functional assay that could detect the reactivity of low frequency antigen specific CD8+ T cells directly from peripheral blood samples [12,13]. We sought to apply this qPCR assay to the semi-automated high throughput functional screening of 96 well plate microcultures containing approximately 150,000 bulk PBMCs and compare these results to our conventional T cell screening evaluation using interferon-γ ELISA of the culture supernatant. In order to determine the sensitivity for both assays in this microwell screening format, we performed a spiking experiment where varying absolute numbers of the C6E4 gp100_154–162 _reactive CD8+ T cell clone were spiked into 150,000 nonreactive autologous bulk PBMCs (Figure 1). No exogenous cytokines were added and culturing of the cells was not performed. The spiked PBMC were immediately tested for their ability to recognize T2 cells pulsed with the relevant gp100_154–162 _peptide (1 uM) and an irrelevant HIV_pol _peptide (1 uM). Cellular interferon-γ mRNA production was measured by qPCR at 3 hours after the co-incubation and supernatant interferon-γ protein production was measured at 24 hours by ELISA. Stimulation indexes (SI) for both assays were determined by dividing the reactivity against the relevant peptide by the reactivity against the irrelevant peptide (SI = gp100_154–162_/HIV_pol_). In the eight replicate wells without spiked C6E4 clone (PBMC alone), neither assay demonstrated significant reactivity (gp100_154–162_/HIV_pol _SI < 2). The qPCR assay did identify T cell reactivity in all replicate wells containing between 3000 and 150 spiked clones (Figure 1A). In at least 2 of the 8 replicate wells, the qPCR assay could detect reactivity at every dilution down to 1.5 cells spiked into 150,000 PBMC. In contrast, the detection limit for interferon-γ protein ELISA was reached in samples with 300 cells spiked into 150,000 PBMC (Figure 1B). We concluded that the qPCR functional assay had a significantly higher sensitivity compared with standard ELISA and it could detect the antigen induced cytokine response of approximately a single CD8+ T cell at precursor frequency of ~1:100,000 in a 96 microwell format.

### qPCR functional screening rapidly identifies melanoma antigen specific T cells in short term sensitized peripheral blood cultures

**Figure 2:**
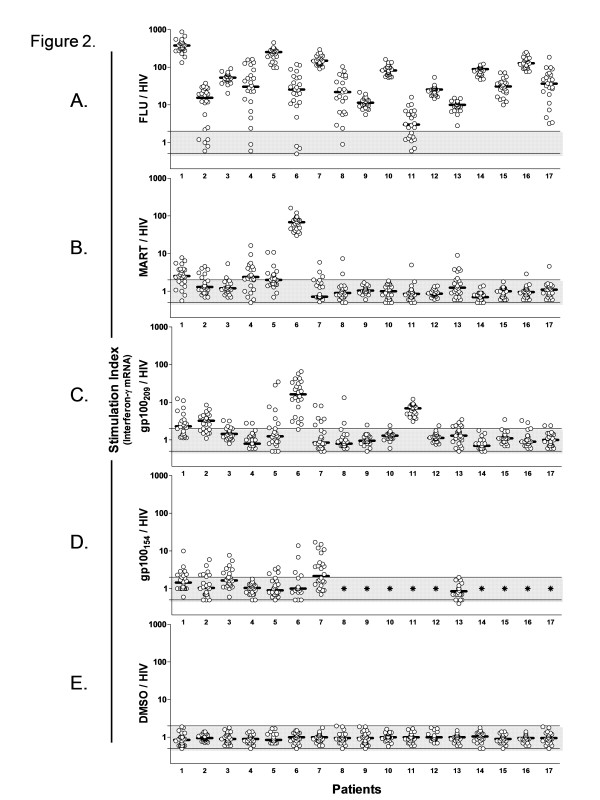
**qPCR functional screening rapidly detects the reactivity of melanoma antigen specific T cells in short term sensitized peripheral blood cultures**. PBMC from 17 HLA-A2+ melanoma patients were plated in replicate microwells (n = 24) containing ~300,000 cells and individually sensitized for 6 days with either 1 μM of (A.) FLUM1, (B.) MART_27–35_, (C.) gp100_209–217_, (D.) gp100_154–162_, or (E.) no peptide (DMSO) in the presence of IL-2 (90 IU/ml). On day 6, a sample from every microculture (~100,000 cells) was screened using the qPCR assay for T cell recognition of the respective sensitizing peptide versus the HIV_pol _peptide pulsed onto T2 cells. Stimulation Index (SI) = IFN-γ mRNA (peptide *x*)/IFN-γ mRNA (HIVpol). (O) represents the SI for each microwell. Bar is median SI value. (*), not done. Shaded area represents range of non-specific reactivity (SI = 0.5–2.0).

We next applied the qPCR assay to the screening of PBMC for natural CD8+ T cell reactivity against known epitopes from the melanocytic differentiation antigens, gp100 and MART. Peripheral blood leukapheresis samples were obtained from 17 HLA-A2+ metastatic melanoma patients who had not previously undergone antigen specific immunotherapy (i.e. vaccine or cell based transfer therapy). Bulk PBMC from each patient were plated in replicate microwells (n = 24) containing ~300,000 cells each and individually sensitized for 6 days with 1 uM of FLUM1, MART_27–35_, gp100_209–217_, gp100_154–162_, or no peptide (DMSO) in the presence of IL-2 (90 IU/ml). On day 6, a sample from every microculture (~100,000 cells) was screened using the qPCR assay for recognition of the respective sensitizing peptide versus the irrelevant HIV_pol _peptide pulsed onto T2 cells (Figure 2). The interferon-γ gene expression was normalized as a SI (peptide *x*/HIV_pol_). The bulk cells cultured in IL-2 with no sensitizing peptide (DMSO alone) were used to define the level of nonspecific background reactivity for each patient (Figure 2E). The median DMSO/HIV_pol _SI for all patients was 1.0 (S.D. ± 0.3) with individual wells ranging from 0.5 to 2.0. By using a cutoff SI value of 2.0, we identified significant microculture reactivity against the FLUM1 peptide in all 17 patients (Figure 2A), which served as an internal positive control for the sensitization procedure. Variability in the median FLUM1/HIV_pol _SI of the replicate wells was observed across patients (median range: 3.0 to 376), consistent with varying degrees of natural peripheral blood CD8+ T cell reactivity against the FLU epitope. Further, despite uniform culture conditions, marked well to well variability within the culture replicates was noted for several patients. Among the cultures sensitized for 6 days with the melanoma antigen epitopes, variable immune reactivity was similarly observed. qPCR analysis of the cultures sensitized with MART_27–35 _(Figure 2B) revealed three patients (Patients 1, 4, and 6) with median MART/HIV SI well reactivity above 2. However, in 12 patients (70.5%), the qPCR assay identified at least one individual microculture replicate which met criteria for significant MART peptide reactivity. Similarly, among the gp100_209–217 _sensitized cultures, only 4 patients (Patients 1, 2, 6, and 11) had median culture reactivity > 2, but 16 of 17 (94%) patients were found to have individual wells with peptide reactivity above background (Figure 2C). Among the 8 patients sensitized with the gp100_154–162 _peptide, one patient (patient 7) had median culture reactivity > 2, but 6 patients (75%) had individual wells with peptide reactivity (Figure 2D). In sum, CD8+ T cell reactivity against at least one of the melanoma epitopes was identified in 16 of the 17 patients (94%). We concluded that the qPCR assay could be used as a highly efficient and rapid screen to detect the reactivity of a variety of melanoma specific T cells in short term sensitized PBMC microcultures.

**Figure 3:**
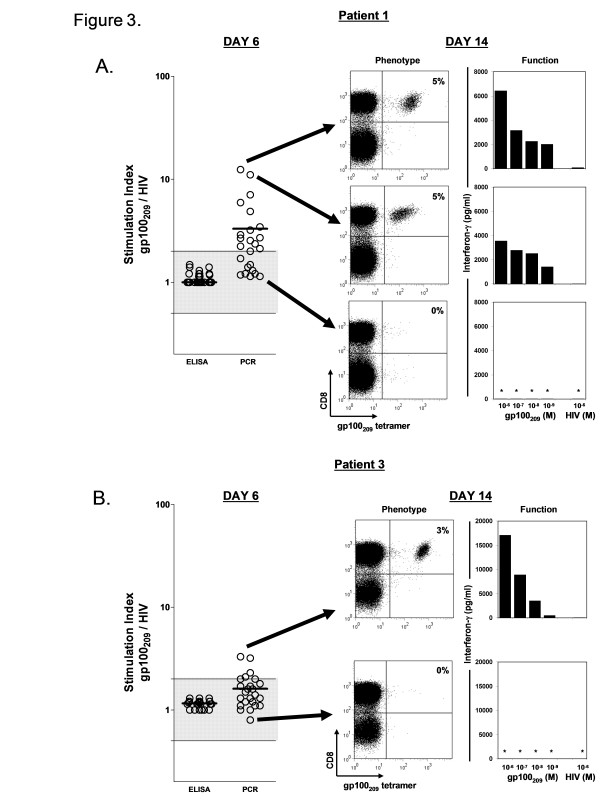
**The magnitude of qPCR reactivity prospectively identifies selected wells enriched for functionally active antigen specific T cells**. Day 6 gp100_209–217 _sensitized microcultures (n = 24) from patients 1 (A.) and 3 (B.) were evaluated in parallel using qPCR and ELISA assays. Significant well reactivity was detected with the qPCR assay, but not with ELISA. Microcultures with the highest and lowest qPCR SIs were selected (see arrow) for rapid expansion. On day 14, the phenotype of the expanded cultures was assessed by staining with gp100_209–217 _peptide/HLA-A*0201 tetramers and anti-CD8 antibody and analysis by flow cytometric analysis. Dot plots are shown for propidium iodide–negative gated cells. Values in FACS dot plots correspond to the percentage of total CD8+ T cells that are tetramer-positive calculated as the number of CD8+ tetramer+ cells divided by the total number of CD8+ T cells minus the CD8- tetramer+ background × 100. Functional reactivity of the expanded cultures was assessed by co-culture with peptide pulsed T2 cells and supernatant analysis by standard ELISA for IFN-γ protein at 24 hrs. ELISA data represents the average of replicate co-culture wells. (O) represents the SI for each microwell. Shaded area represents range of non-specific reactivity (SI = 0.5–2.0). (*), not detectable.

To determine whether the immune reactivity identified at day 6 by the qPCR assay could also be detected by ELISA, gp100_209–217 _sensitized microcultures from patients 1 and 3 were evaluated using both assays with an equivalent number of sampled PBMC (~100,000 cells) from each of the replicate wells (Figure 3A and 3B). ELISA evaluation did not identify any wells from either patient with reactivity above background. In contrast the qPCR assay performed on the same wells demonstrated multiple cultures with detectable peptide reactivity. To confirm that the qPCR reactivity in these early cultures independently correlated with the presence of gp100_209–217 _specific T cells, the microcultures with the highest and lowest SIs were rapidly expanded with anti-CD3, allogenic feeder cells, and IL-2 over 1 week and evaluated for the presence and activity of gp100_209–217 _reactive CD8+ T cells (Figure 3A and 3B). By day 14, the expanded cultures from the wells with the high SI (Patient 1, SI = 11.1 and 12.4; Patient 3, SI = 3.3) demonstrated a distinct population of antigen specific CD8+ T cells when stained with the gp100_209–217 _tetramer (3–5% of CD8+ cells). When samples of these expanded cultures were tested for functional recognition of T2 cells pulsed with the gp100_209–217 _peptide, they released significant amounts of interferon-γ protein that could now easily be detected by ELISA. In contrast, the expanded cultures from the low SI wells (Patient 1, SI = 1.1; Patient 3, SI = 0.8) had neither discernable tetramer positive cells nor functional activity against peptide pulsed targets. We concluded that the qPCR assay could be used at an early time point to stratify the epitope reactivity of short term sensitized PBMC microcultures to prospectively identify selected wells enriched for functionally active antigen specific T cells and to identify wells with no evidence of reactivity.

### A novel strategy for high throughput isolation of low frequency melanoma specific T cell clones from peripheral blood

**Figure 4:**
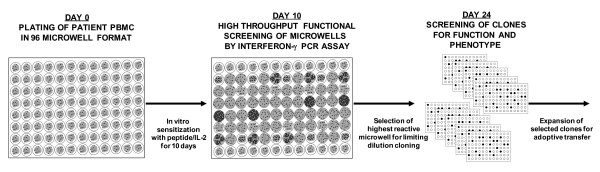
Strategy for high throughput cloning of low frequency antigen specific T cells from peripheral blood for adoptive immunotherapy.

**Figure 5:**
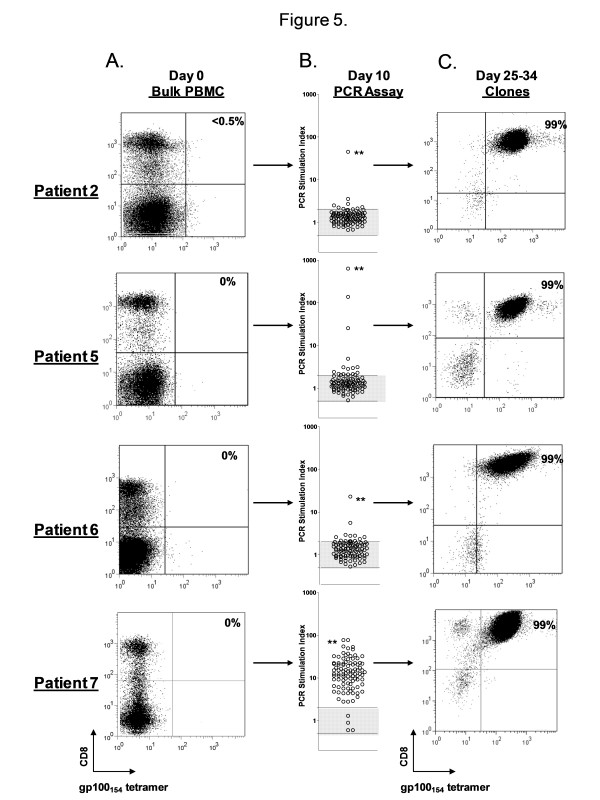
**Rapid cloning of gp100_154–162 _specific CD8+ T cells from peripheral blood**. (A.) On Day 0, PBMC from four HLA-A2+ melanoma patients underwent staining with gp100_154–162 _peptide/HLA-A*0201 tetramers and anti-CD8 APC to determine natural precursor frequency. None of the patients demonstrated a significant population of tetramer positive CD8+ T cells by FACS. PBMC from each patient were plated in replicate microwells (n = 96) containing ~300,000 cells and sensitized for 10 days with 1 μM of gp100_154–162 _peptide in the presence of IL-2 (90 IU/ml). (B.) On day 10, a sample from every microculture was screened using the qPCR assay for specific recognition of the gp100_154–162 _peptide versus the HIV_pol _peptide. The wells with the highest SI reactivity (denoted by **) were selected for limiting dilution cloning. After approximately 2 weeks, growth positive wells were functional screened for their ability to lyse peptide pulsed T2 cells. Selected T cell clones were expanded and underwent FACS analysis (C.) between days 25 and 34 to reveal highly enriched (99%) populations of gp100_154–162 _tetramer positive CD8+ T cells. Values in FACS dot plots correspond to the percentage of total CD8 + T cells that are tetramer-positive. (O) represents the SI for each microwell. Shaded area represents range of non-specific reactivity (SI = 0.5–2.0).

**Figure 6:**
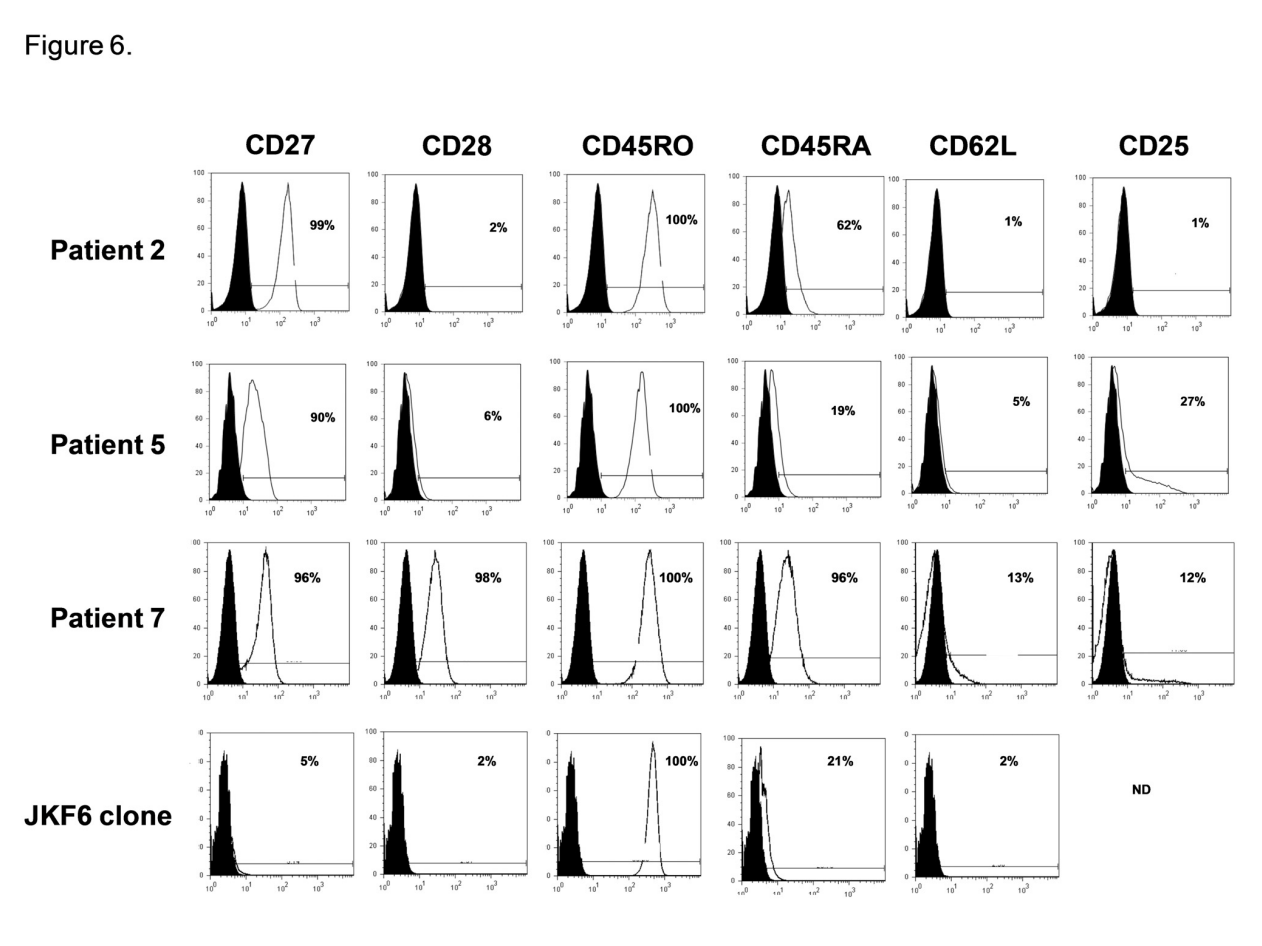
**Phenotype of gp100_154–162 _specific CD8+ T cell clones**. Peripheral blood T cell clones underwent staining with gp100_154–162 _peptide/HLA-A*0201 tetramers and anti-CD8. The CD8+/tetramer+ cells were assessed for cell surface expression of CD27, CD28, CD45RO, CD45RA, CD62L, and CD25. Analysis was performed with multiparameter flow cytometry. Values on histograms represent the percentage of total CD8+ T cells that are tetramer-positive and express the indicated marker. JKF6 is a MART_27–35 _specific CD8+ T cell clone derived from TIL. ND, not done.

We next sought to incorporate the qPCR functional assay into a novel strategy for high throughput isolation of low frequency antigen specific CD8+ T cell clones from bulk PBMC for use in adoptive immunotherapy trials. We initially aimed to isolate CD8+ T cell clones reactive against the gp100_154–162 _epitope given their extremely low natural frequency in peripheral blood, the difficulty in prospectively isolating these T cells, and the limited published reports of T cell clones reactive against this epitope [7]. The basic isolation strategy is summarized in Figure 4. PBMC from an HLA-A2+ patient with metastatic melanoma are used to establish 96 independent microcultures which are sensitized for 10 days with 1 uM of gp100_154–162 _in the presence of IL-2 (90 IU/ml). On day 10, a sample from every microculture is screened using the qPCR assay for specific recognition of the gp100_154–162 _peptide versus the HIV_pol _peptide. The SI reactivities for the 96 wells are stratified by their magnitude and the most reactive microcultures are selected for the next step, limiting dilution cloning. After approximately 2 weeks, growth positive wells are screened for their ability to lyse peptide pulsed T2 cells and melanoma tumor lines. Selected T cell clones are then expanded in the final step for further analysis and potential use in adoptive immunotherapy trials. This strategy was applied to PBMC from four melanoma patients (patients 2, 5, 6, and 7) (Figure 5). A sample of the bulk PBMC from each patient, prior to any *in vitro *manipulation underwent staining with the gp100_154–162 _tetramer to determine natural precursor frequency. None of the patients demonstrated a significant population of tetramer positive CD8+ T cells by FACS on day 0 (Figure 5A). After 10 days of sensitization, the 96 independent microcultures for each patient were screened for peptide reactivity using the qPCR assay (Figure 5B). The stratified results for Patients 2, 5, and 6 demonstrated that only 7%, 12%, and 8% of the wells had a SI ≥ 2, respectively; 1%, 3%, and 1% of the wells had a SI ≥ 10, respectively; and the remaining wells had no detectable peptide reactivity (SI < 2). In contrast, for Patient 7, 92% of the wells had a SI ≥ 2 and 60% of the wells had a SI ≥ 10. The highest reactive microcultures from patients 2, 5, 6, and 7 (qPCR SI = 45, 635, 23, and 78, respectively) were selected for limiting dilution cloning. The frequencies of growth positive clones with lytic ability against peptide pulsed targets were 0.2%, 28%, 0.1%, and 2.3% for the respective patients, which directly correlated with the qPCR SI (r^2 ^= 0.99, p < 0.0001). These selected clones were expanded and underwent FACS analysis between days 25 and 34 to reveal highly enriched populations (99%) of gp100_154–162 _tetramer positive CD8+ T cells (Figure 5C). Further, the derived populations were confirmed to be clonal by the sequencing of a single T cell receptor Vα and Vβ chain for each patient (Table 1). The functional avidity of these isolated T cell clones was high, as measured by their ability to recognize 10^-10 ^to 10^-11 ^M of gp100_154–162 _peptide pulsed onto T2 cells and HLA A2+/gp100+ melanoma tumor lines *in vitro *(Table 2). The phenotype of these cells was assessed by cell surface FACS for CD27, CD28, CD45RO, CD45RA, CD62L, and CD25 (Figure 6). The gp100_154–162 _tetramer positive cells from patients 2, 5, and 7 all were uniformly CD45RO+ and CD62L-, consistent with an effector memory phenotype. However, unlike typical antigen experienced T cells, there was persistent variable expression of CD45RA (19–96%). In addition all of the isolated clones continued to have significant expression of the costimulatory molecule CD27 (90–99%). This phenotype differed from the terminally differentiated TIL derived MART_27–35 _specific clone, JKF6, which had no significant expression of CD27. Since initiating these isolation studies we have successfully cloned gp100_154–162 _CD8+ T cells in 6 of 8 patients (75%). In pilot clinical scale expansions, these clones demonstrated between 850–1000 fold expansions in cell numbers over the initial 14 days after a single rapid expansion in flasks. A second serial expansion of these clones resulted in an additional 400–600 fold expansion over the ensuing week. Thus, we found that with two consecutive rapid expansions ~10^10 ^cells could be generated for potential clinical adoptive transfer from each starting clone isolated.

**Table 1:** TCR α/β Complementarity Determining Region Residues Identified from gp100_154–162 _specific CD8+ T cell clones

		**α-CDR1**						**α-CDR2**								**α-CDR3**											
	**TRAV**																										

Patient 2	12.1	N	S	A	S	Q	S		V	Y	S	S	G			V	V	N	M	N	S	N	Y	Q	L	I	

Patient 5	8.1	Y	G	G	T	V	N		Y	F	S	G	D	P	L		A	V	N	G	D	D	K	I	I		

Patient 6	5	D	S	S	S	T	Y		I	F	S	N	M	D		A	E	M	A	N	S	G	Y	A	L	N	

Patient 7	5	D	S	S	S	T	Y		I	F	S	N	M	D		A	E	N	P	E	G	N	D	M	R		

		**β-CDR1**						**β-CDR2**							**β-CDR3**												

	**TRBV**																										

Patient 2	15	L	N	H	N	V		Y	Y	D	K	D	F		A	T	S	Q	E	A	S	F	T	D	T	Q	Y

Patient 5	7.6	S	G	H	V	S		F	N	Y	E	A	Q		A	S	S	L	G	G	G	Q	D	T	Q	Y	

Patient 6	12.3	S	G	H	N	S		F	N	N	N	V	P		A	S	S	P	G	G	G	E	Q	F			

Patient 7	11.2	S	G	H	A	T		F	Q	N	N	G	V		A	S	S	P	G	G	G	G	E	Q	F		

**Table 2:** Peptide and tumor reactivity of gp100_154–162 _specific CD8+ T cell clones *

	**Peptide Reactivity**								**Tumor Reactivity**				
	**gp100_154 _(M)**							**HIV (M)**	**A2+/gp100+**	**A2+/gp100+**	**A2-/gp100+**	**A2-/gp100-**	
	**10^-6^**	**10^-7^**	**10^-8^**	**10^-9^**	**10^-10^**	**10^-11^**	**10^-12^**	**10^-6^**	**Mel 526**	**Mel 624**	**Mel 888**	**Hep 3B**	**Media**

**Patient 2 clone**	**7105**	**6606**	**5102**	**2555**	**187**	< 10	< 10	< 10	**2724**	**5743**	< 10	< 10	< 10

**Patient 5 clone**	**12067**	**11274**	**5323**	**1210**	**244**	< 10	< 10	< 10	**5114**	**7425**	< 10	< 10	< 10

**Patient 7 clone**	**33349**	**29191**	**26858**	**18575**	**687**	**220**	< 10	< 10	**3975**	**8434**	< 10	< 10	< 10

* Specific reactivity of cloned T cells was evaluated by tumor or peptide-specific stimulation. 1e5 cloned T cells were cocultured overnight with an equal number of melanoma tumor cells or T2 cells pulsed with either 1 μM irrelevant HIV peptide or titered concentrations of gp100:154–162 peptide, and assessed for IFN-g (pg/ml) production by standard ELISA

Values 100 pg/ml and twice background are bolded and underlined.

## Discussion

Cancer immunotherapy by means of adoptive transfer of antigen specific T cells has several conceptual advantages. First, lymphocytes with high avidity against specific tumor antigens can be selected. Next, the lymphocytes can be activated *ex-vivo *to exhibit anti-tumor effector function and expanded to large numbers for administration. Finally, the host may be manipulated to facilitate the function and persistence of the transferred cells. The evidence that this immunotherapy approach can mediate significant tumor regression comes from clinical studies transferring *ex vivo *expanded TIL to lymphodepleted metastatic melanoma patients [1,2]. However, given the polyclonal nature of bulk TIL, it has been difficult from these early studies to define a putative "optimal" T cell population that can predictably result in tumor regression. Analyses of pre-clinical and clinical studies of adoptive immunotherapy have suggested potential lymphocyte characteristics associated with improved outcome. Murine studies of adoptive transfer have demonstrated that high avidity CTL possess superior in vivo anti-viral and anti-tumor efficacy when compared to low avidity CTL [14,15](In the *pmel *murine model of melanoma, adoptive transfer of CTL with terminal effector function and phenotype induced by repetitive *in vitro *antigen and IL-2 stimulation was associated with impaired *in vivo *anti-tumor efficacy [16]. Thus, the adoptive transfer of less differentiated antigen specific cells with an early effector memory phenotype was advocated. Retrospective evaluation of human trials administering melanoma reactive TIL have suggested that longer telomere length of bulk TIL correlated with *in vivo *persistence and tumor regression in melanoma patients receiving cell transfer [17]. Further, the number of CD27+/CD8+ T cells in bulk TIL was associated with the ability of these TIL to mediate tumor regression following adoptive transfer [18]. With respect to lymphocyte specificity, an optimal antigenic target for the immunotherapy of melanoma has not been clearly established in human trials. Given the many significant variables that might influence clinical tumor response, we hypothesized that an iterative strategy of isolation, transfer, and clinical evaluation of a series of differing autologous tumor reactive lymphocyte clones would help to define the antigenic targets and lymphocyte characteristics associated with therapeutic efficacy.

Critical to this approach is the ability to identify, isolate and expand a variety of self reactive T cell clones that exist at low frequencies in the natural T cell repertoire. Therefore, to conduct these investigations, we developed a novel *in vitro *cloning methodology using quantitative RT-PCR (qPCR) as a highly sensitive functional screen to detect the reactivity of low frequency T cells in the peripheral blood of metastatic cancer patients. We determined that the interferon-γ qPCR assay was capable of detecting the reactivity of 1 antigen specific T cell in approximately 100,000 background cells. This sensitivity was shown to be superior to our conventional screening using interferon-γ ELISA and also compared favorably to other published functional assays such as intracellular cytokine FACS (1:10,000 cells), cytometric cytokine capture assays (1:10,000 cells), and enzyme-linked immunospot (ELISPOT) (1:50,000 cells) [19].

The use of peptide/MHC multimers and FACS sorting has previously been suggested as an effective means of T cell enrichment for rapid cloning [7,20], however, this technique has generally been difficult to apply to many tumor-specific lymphocytes because of their low precursor frequency and the sensitivity limitations of FACS analysis. Furthermore, peptide/MHC complexes are capable of measuring the presence of lymphocytes possessing the appropriate T cell receptor but not the functional state or the ability of these lymphocytes to respond to exposure to tumor antigen. In our studies, the qPCR assay could detect the functional reactivity of T cells against a variety of known melanoma tumor epitopes (MART_27–35_, gp100_209–217_, gp100_154–162_) in short term sensitized microcultures (Figure 2). Further, the magnitude of the epitope reactivity could be used to prospectively select individual cultures with the greatest enrichment for functionally active antigen specific T cells and, thus, eliminate the majority of non reactive cultures after approximately one week. This early high throughput screening step combined with immediate limiting dilution cloning of highly selected microcultures has allowed for the rapid and efficient isolation of rare antigen specific T cell clones and has eliminated the prolonged culture times typically required for their generation. In this report, we utilized this strategy to clone gp100_154–162 _reactive lymphocytes from the natural peripheral blood repertoire of four melanoma patients, a process that has previously been exceedingly difficult and time consuming with conventional repetitive *in vitro *stimulation techniques [21,22]. In addition to improved efficiency of isolation, qualitative evaluation of the lymphocytes cloned with this technique has demonstrated several favorable characteristics. The gp100_154–162 _reactive clones were highly avid and could recognize between 10^-10 ^to 10^-11 ^M of gp100_154–162 _peptide pulsed onto T2 cells, as well as, antigen naturally processed and presented by melanoma tumor lines. The lymphocyte cultures were generated in the setting of limited peptide exposure (~10 days) and low concentrations of IL-2 (90 IU/ml), conditions suggested as beneficial for the *in vivo *efficacy of adoptively transferred T cells [16]. The expanded clones had an effector memory phenotype with high cell surface expression of CD27, an attribute that was associated with tumor regression with TIL therapy [18]. Finally, these cells showed significant proliferative capacity to reach cell numbers appropriate for clinical trials (~10^10 ^cells). For these reasons, we believe the lymphocytes cloned using this strategy represent a unique and attractive population of cells for adoptive immunotherapy that differ from previously administered clones generated with prolonged and repetitive in vitro stimulation which have had minimal therapeutic efficacy [5,6,10,11]. Plans are underway to clinically transfer gp100_154–162 _reactive clones in conjunction with a lymphodepleting conditioning chemotherapy regimen to patients with refractory melanoma.

An additional important aspect to our methodology is the application of high throughput robotic automation. We currently perform all steps involved in the screening and isolation process with automated 96 well liquid dispensing instruments which have enabled the isolation of T cell clones in an extremely time efficient manner, a critical requirement for metastatic cancer patients waiting for therapy. We currently are exploring the adaptation of this method to a 384 well automated format which would further increase the throughput and efficiency of the cloning procedure. Finally, the basic platform that we have described in this report can be applied to the rapid isolation of virtually any antigen specific T cell population (CD4+ or CD8+) where the immunogenic epitope is known. We are currently utilizing this methodology for the isolation of other tumor and viral specific lymphocytes for future adoptive immunotherapy trials and are also extrapolating this methodology to the rapid screening and evaluation of novel epitope targets as means of tumor antigen/epitope discovery.

## Conclusion

This report describes a novel high efficiency strategy to clone tumor reactive T cells from peripheral blood for use in adoptive immunotherapy. We are currently utilizing this methodology for the isolation of other tumor and viral specific lymphocytes for future adoptive immunotherapy trials and are also extrapolating this methodology to the rapid screening and evaluation of novel epitope targets as means of tumor antigen/epitope discovery.

## Competing interests

The authors declare that they have no competing interests.

## Authors' contributions

USK conceived of the study and participated in its design, execution and helped to draft the manuscript. OSK executed critical experiments and helped to draft the manuscript. All authors read and approved the final manuscript.
